# A mutational signature in gastric cancer suggests therapeutic strategies

**DOI:** 10.1038/ncomms9683

**Published:** 2015-10-29

**Authors:** Ludmil B. Alexandrov, Serena Nik-Zainal, Hoi Cheong Siu, Suet Yi Leung, Michael R Stratton

**Affiliations:** 1 Cancer Genome Project, Wellcome Trust Sanger Institute, Hinxton, Cambridgeshire CB10 1SA, UK; 2 Theoretical Biology and Biophysics (T-6), Los Alamos National Laboratory, Los Alamos, New Mexico 87545, USA; 3 Center for Nonlinear Studies, Los Alamos National Laboratory, Los Alamos, New Mexico 87545, USA; 4 Department of Medical Genetics, Addenbrooke's Hospital National Health Service (NHS) Trust, Cambridge CB2 0QQ, UK; 5 Department of Pathology, The University of Hong Kong, Queen Mary Hospital, Pokfulam, Hong Kong

## Abstract

Targeting defects in the DNA repair machinery of neoplastic cells, for example, those due to inactivating *BRCA1* and/or *BRCA2* mutations, has been used for developing new therapies in certain types of breast, ovarian and pancreatic cancers. Recently, a mutational signature was associated with failure of double-strand DNA break repair by homologous recombination based on its high mutational burden in samples harbouring *BRCA1* or *BRCA2* mutations. In pancreatic cancer, all responders to platinum therapy exhibit this mutational signature including a sample that lacked any defects in *BRCA1* or *BRCA2*. Here, we examine 10,250 cancer genomes across 36 types of cancer and demonstrate that, in addition to breast, ovarian and pancreatic cancers, gastric cancer is another cancer type that exhibits this mutational signature. Our results suggest that 7–12% of gastric cancers have defective double-strand DNA break repair by homologous recombination and may benefit from either platinum therapy or PARP inhibitors.

## Abstract (web summary)

Cancer genome analysis has demonstrated that some breast and ovarian tumours show reduced homologous recombination, a feature that can be therapeutically exploited. Here, Alexandrov *et al.* search for this mutational signature in 36 different cancer types and find that some gastric tumours also harbour this mutational spectrum.

## Graphical abstract

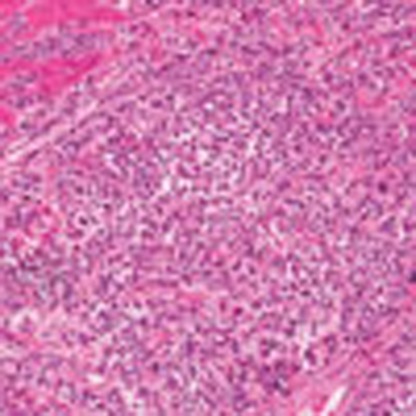

Germline inactivating mutations in *BRCA1* and/or *BRCA2* cause an increased risk of early-onset breast^1^,^2^, ovarian^2^,^3^ and pancreatic cancer^4^, while somatic mutations in these two genes and *BRCA1* promoter hypermethylation have also been implicated in development of these cancer types^5^,^6^. *BRCA1* and *BRCA2* are involved in error-free homology directed double-strand break repair^7^. Cancers with defects in *BRCA1* and *BRCA2* consequently show large numbers of rearrangements and indels due to error-prone repair by non-homologous end joining mechanisms, which assume responsibility for double-strand break repair^8^,^9^.

While defective double-strand break repair increases the mutational burden of a cell, thus increasing the chances of acquiring somatic mutations that lead to neoplastic transformation, it also renders a cell more susceptible to cell cycle arrest and subsequent apoptosis when it is exposed to agents such as platinum-based antineoplastic drugs^10^,^11^. This susceptibility has been successfully leveraged for the development of targeted and less-toxic therapeutic strategies for treatment of breast, ovarian and pancreatic cancers harbouring *BRCA1* and/or *BRCA2* mutations, notably poly(adenosine diphosphate ribose) polymerase (PARP) inhibitors^10^,^11^. These treatments cause a multitude of DNA double-strand breaks that force neoplastic cells with defective *BRCA1* and *BRCA2* function into apoptosis since they lack the ability to effectively repair double-strand breaks. In contrast, normal cells remain mostly unaffected since their repair machinery is not compromised.

Exposure to exogenous or endogenous mutagens, abnormal DNA editing, the incomplete fidelity of DNA polymerases and failure of DNA repair mechanisms generate distinct combinations of somatic mutation types in cancer genomes^12^,^13^,^14^. We previously termed such patterns ‘mutational signatures' and developed an approach for extracting mutational signatures from cancer genomes^15^. Our previous analysis revealed 21 distinct base-substitution signatures across the spectrum of human cancer^12^. These base-substitution mutational signatures were described using a simple classification based on the six classes of single-base mutations: C>A, C>G, C>T, T>A, T>C and T>G (all substitutions are referred to by the pyrimidine of the mutated Watson–Crick base pair) in combination with the base immediately 5′ and 3′ to each mutation, thus resulting in 96 possible mutation types. Our previous analysis^12^ revealed that cancers harbouring germline and/or somatic mutations in *BRCA1* and *BRCA2* exhibited a specific base-substitution signature, termed signature 3. The mutational pattern of signature 3 is shown in Fig. 1a. Interestingly, although almost all breast, ovarian and pancreatic cancers with *BRCA1/2* mutations have large numbers of signature 3 mutations, a number of cancer cases lacking mutations in *BRCA1* and *BRCA2* or other genes known to play a role in double-strand break repair also exhibit the mutational signature^12^. This observation hinted towards the existence of other mechanisms that may be disabling homology directed double-strand DNA break repair.

A recent clinical analysis of pancreatic whole-genomesequencing data revealed that all samples responding to platinum therapy exhibited substantial numbers of signature 3 mutations^16^. This therapeutic response was also observed in a sample that lacked any germline or somatic *BRCA1* or *BRCA2* mutations, indicating that signature 3 itself could be used for decision support in allocating these therapies, even in the absence of *BRCA1* or *BRCA2* mutations.

In this study, we report a large-scale mutational signatures analysis aiming to identify the presence of signature 3 across human neoplasia. Our results reveal that, in addition to previously known cancer types, signature 3 is also present in 7–12% of gastric cancers. These gastric cancers most likely have defective homology directed double-strand DNA break repair and may benefit from either platinum therapy or PARP inhibitors.

## Results

### Large-scale survey of signature 3 across human neoplasia

**Figure 1:**
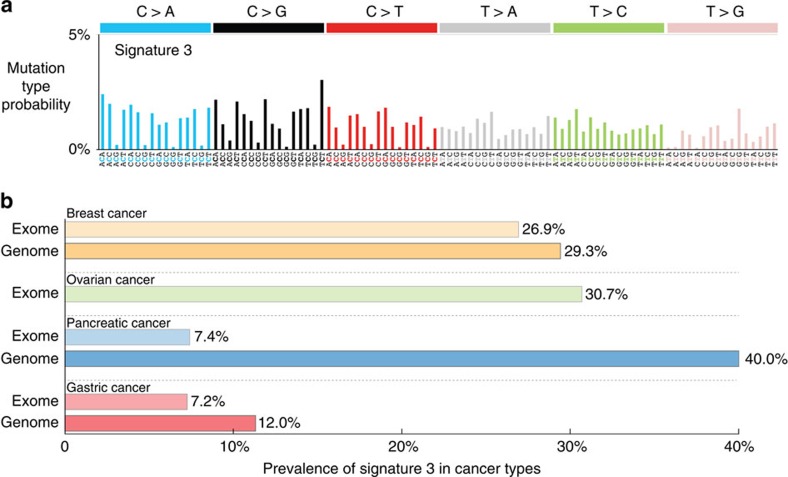
Signature 3 and its presence in human cancer. (**a**) The mutational pattern of signature 3. The signature is displayed according to the 96 substitution classification defined by the substitution class and sequence context immediately 5′ and 3′ to the mutated base. The probability bars for the six substitution classes are displayed in different colours. The mutation subtypes are on the *x* axis, and the *y* axis shows the percentage of mutations in the signature attributed to each mutation type displayed on the basis of the trinucleotide frequencies of the whole human genome. (**b**) Prevalence of signature 3 across human cancer types. The *x* axis depicts the percentage of samples in which signature 3 was observed. The *y* axis reflects the cancer types in which signature 3 was observed as well as whether the data were derived via whole-genome or whole-exome sequencing. Note that the data set did not have any ovarian whole-genome sequenced cancers. Further, it should be noted that the pancreatic whole-genome sequenced samples were deliberately enriched with *BRCA1/2* mutations explaining the high prevalence of signature 3.

We used a substantially elaborated version of our previously developed framework for deciphering mutational signatures (Methods) and analysed 7,329,860 somatic mutations from 10,250 pairs of cancer-normal samples derived from 36 distinct types of human cancer, including 607 whole-genome sequences and 9,643 whole-exome sequences (Supplementary Data 1). As expected, signature 3 was found in ovarian, breast and pancreatic cancers (Fig. 1b; Supplementary Data 1). In ovarian cancer 143 of the examined 466 ovarian whole exomes (∼30.7% of ovarian samples) exhibited signature 3. In breast cancer, signature 3 was found in 283 of the 1,051 whole-exome breast cancer sequences (∼26.9%) and in 35 of the 119 whole-genome sequences (∼29.4%). Whole-genome sequencing of 15 pancreatic cancers deliberately enriched for cases with *BRCA1/2* mutations revealed the presence of signature 3 in six samples (40.0%), while examination of an unbiased set of 216 whole-exome sequenced pancreatic cancers identified signature 3 in 16 cases (∼7.41%).

**Figure 2:**
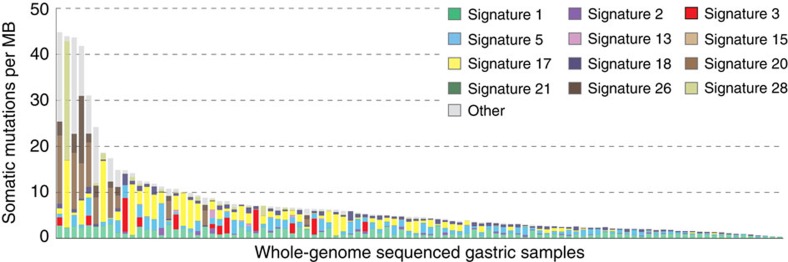
The contributions of mutational signatures to individual gastric cancer whole-genome sequenced samples. Each bar represents a whole-genome sequenced gastric cancer sample and is coloured proportionally to the number of somatic mutations contributed by each mutational signature. The vertical axis denotes number of mutations per megabase. Signature 3 is coloured in red for clarity. ‘Other' refers to mutational signatures that have not been previously validated.

**Figure 3:**
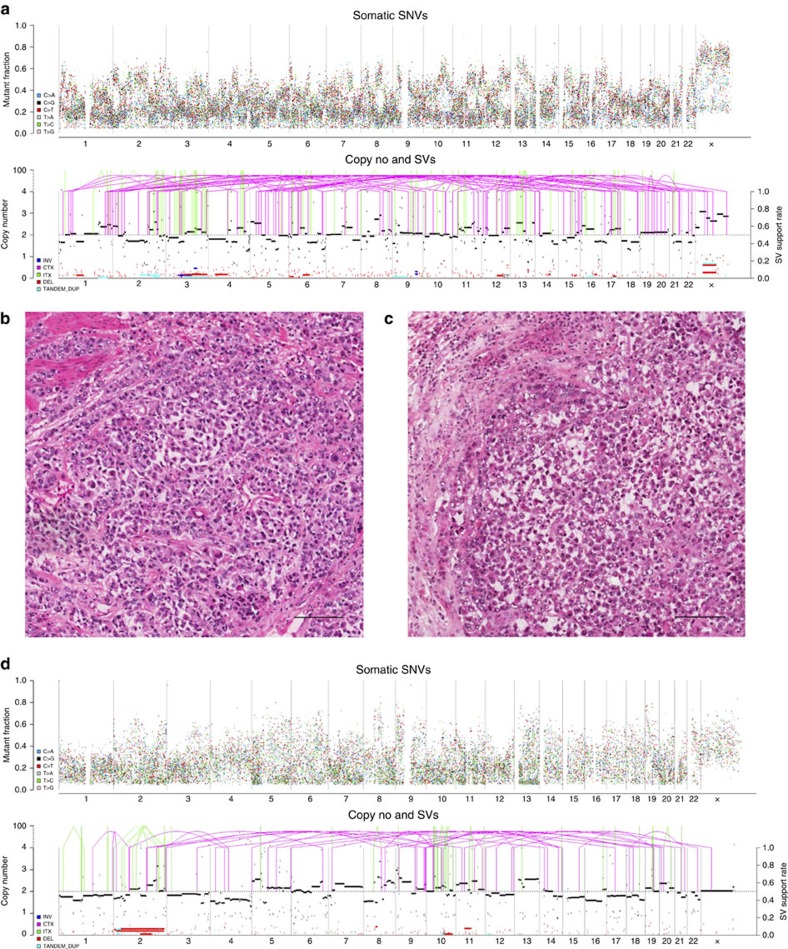
Two representative examples of gastric cancers harbouring signature 3 and their corresponding histology and genomic alterations. (**a**,**b**) A gastric cancer with a *BRCA2* somatic mutation (that is, pfg053T); (**c**,**d**) another gastric cancer without any known *BRCA1* or *BRCA2* mutations (that is, pfg034T). In sections (**a**,**d**), the horizontal axes indicate chromosomal positions in ascending order. The top panel indicates somatic substitutions, each dot represents a somatic mutation with their mutant fraction shown in the vertical axis. The bottom panel indicates variation in chromosome copy number and structural variants. (**b**,**c**) Haematoxylin and eosin sections of gastric cancers, both showing compact sheets of malignant cells growing in marked discohesive pattern. Scale bar, 100 μm.

Remarkably, despite surveying another 33 distinct cancer types derived from diverse epithelial, mesenchymal, glial, haematopoietic and lymphoid cells, signature 3 was observed only in gastric cancer. The examined data for gastric cancer included 372 whole-exome and 100 whole-genome sequences (Supplementary Data 1). These data were derived from four independent previously published studies^17^,^18^,^19^,^20^. We were able to detect signature 3 in 27 whole exomes (∼7.3% of the examined whole-exome gastric samples) and in 12 whole-genomes (12.0% of the examined whole-genome gastric samples). Some gastric samples harboured *BRCA1* or *BRCA2* somatic mutations, but there was no enrichment of signature 3 in samples with *BRCA1*/2 mutations. Most of these *BRCA1/2* mutations were heterozygous and were found in cases with a very high prevalence of small indels and base substitutions due to defective DNA mismatch repair and are, therefore, highly likely to be passenger mutations. The contributions of all mutational signatures operative in the examined set of gastric cancers are provided in Supplementary Data 2, while the mutational signatures in the whole-genome-sequenced samples are shown in Fig. 2. In addition, the genomic profiles of two gastric samples harbouring signature 3 (one sample with a *BRCA2* mutation and another without a mutation in either *BRCA1* or *BRCA2*) are shown in Fig. 3.

### Patterns of indels and structural rearrangements

The presence of signature 3 mutations (and thus failure of DNA double-strand repair by homologus recombination) is closely associated with a particular pattern of large indels (longer than three base pairs) with overlapping microhomology at the deletion break points in breast, ovarian and pancreatic cancers. This pattern provides additional evidence of the absence of homologous recombination-based repair and the role of non-homologous end-joining mechanisms. To evaluate further the significance of finding signature 3 mutations in gastric cancer, we searched for indels of this type. Whole-genome sequenced gastric cancers harbouring signature 3 had a median number of indels with overlapping microhomologies at break points of 715 compared with a median number of only 172 such indels in samples in which there was no evidence for signature 3 (Supplementary Data 3; Mann–Whitney *U*-test's *P* value=1.07 × 10^−5^). Similarly, gastric whole-exomes exhibited a statistically significant elevation of large indels with overlapping microhomologies in samples in which signature 3 was found to be operative (Supplementary Data 4; Mann–Whitney *U*-test's *P* value=5.87 × 10^−4^).

Breast, ovarian and pancreatic cancers with *BRCA1* or *BRCA2* mutations and/or signature 3 mutations show larger numbers of structural rearrangements than cases without, consistent with a deficiency in error-free double-strand break repair. We therefore compared the numbers of rearrangements in gastric cancers with and without signature 3 mutations. Whole-genome sequenced samples harbouring signature 3 had on average 244 structural variants versus 111 in samples that did not exhibit signature 3 (Fig. 2; Supplementary Data 3; Mann–Whitney *U*-test's *P* value=1.24 × 10^−3^). Thus, gastric cancers with signature 3 mutations bear other mutational hallmarks of failure of homology directed double-strand break repair, despite the absence of inactivating mutations in *BRCA1* and *BRCA2* genes.

### Association between signature 3 and gastric cancer histology

Examining the histology of gastric samples with signature 3 revealed that they are enriched for the intestinal type by Lauren's classification (Table 1; Fisher's exact test's *P* value=5.80 × 10^−3^) and have a tendency to display a distinctive compact discohesive growth pattern that looks like ‘growth in cell suspension' (Fig. 3b,c; Table 1; Fisher's exact test's *P* value=3.00 × 10^−4^). This pattern is characterized by solid nests of roundish malignant cells with marked loss in cell-to-cell adhesion (Fig. 3b,c). These nests are crowded together, which distinguishes them from the diffuse-type gastric cancer with widely infiltrative growth behaviour.

### Presence of signature 3 in gastric cell lines

Our analysis of 10,250 primary cancers was also complemented by examination of the generally available set of gastric cell lines to provide a suitable model for testing the drug susceptibility of stomach cancers harbouring signature 3. In total, we examined whole-exome sequences of 20 gastric cell lines (Supplementary Data 5). Unfortunately, we were not able to identify a cell line in which signature 3 was present. This is perhaps unsurprising since the 20 cell lines lacked a matched-normal control, thus complicating the detection of mutational signatures due to contamination with high numbers of private germline polymorphisms.

## Discussion

The results of this study provide the first comprehensive large-scale survey of mutational signature 3 across human cancer. It should be noted that most of the analysed 10,250 samples were whole-exome sequenced (94% of samples) and it is possible that our survey was not able to detect the presence of signature 3 in cancer types and samples in which the signature generates low numbers of somatic mutations. Nevertheless, our analysis demonstrates that signature 3 is present in ∼7% of whole-exome sequenced gastric cancers as well as 12% of whole-genome sequenced gastric cancers. The results indicate that stomach cancers with signature 3 mutations may have defective homology directed double-strand DNA break repair.

Only very limited clinical data were available for the examined gastric cancers restricting our opportunities for exploring correlations between the presence of signature 3 with disease progressions and outcome following treatment. Future studies, involving larger cohorts of samples with carefully curated clinically based data coupled with detailed histological data and complimented by functional analysis, will be necessary to further elaborate the connection between signature 3 and clinical response in gastric cancer.

Gastric cancer is the second most common cause of cancer-related deaths worldwide^21^. Since cancers with defective homology directed double-strand break repair due to *BRCA1* or *BRCA2* mutations are particularly sensitive to platinum therapy and PARP inhibitors, it is conceivable that this subset of gastric cancers might also benefit from their usage. Current gastric cancer chemotherapy protocols are variable and may include 5-fluorouracil-based therapy only or in combination with platinum-based drugs and other agents. Future precision medicine clinical studies with a focus on patient selection will be required to evaluate whether the presence of signature 3 substitutions, and the features of indels and rearrangements associated with it, might allow better patient selection for platinum-based drugs, and whether targeted therapies such as PARP inhibitors based on defective DNA double-strand break repair would also benefit patients with gastric cancer.

## Methods

### Curation of freely available somatic mutations of cancer samples

No data were generated specifically for the uses of this study. Rather, a large-scale data curation was performed with the goal of annotating the majority of freely available cancer genomes. Somatic mutations identified in 10,250 genome pairs (consisting of a cancer genome and the genome of a matched normal tissue) were curated. The curated data encompass 36 distinct types of cancer. In all, 607 of the 10,250 matched-normal pairs had their whole-genome sequenced, while the remaining 9,643 were subjected to whole-exome sequencing. Data were retrieved from three main sources: (i) the data portal of The Cancer Genome Atlas, (ii) the data portal of the International Cancer Genome Consortium and (iii) previously published data in peer-reviewed journals. Information for each sample, including its original data source, is provided in Supplementary Data 1. The somatic mutations for all examined samples are freely available and can be retrieved based on the information provided in Supplementary Data 1.

### Filtering of somatic mutations and generating mutational catalogues

This study relies on previously sequenced samples from cancer and normal tissues, as well as from the subsequently used bioinformatics analyses to identify cancer tissue-specific somatic mutations. The analysed sequencing data were originally generated by a variety of different laboratories, leveraging different sequencing platforms and using a diverse set of mutation-calling algorithms. To remove any residual germline mutations as well as technology-, institute- and/or laboratory-specific sequencing artefacts, extensive filtering was performed before analysing the data. Germline mutations were filtered out from the lists of reported somatic mutations using the complete list of germline mutations from dbSNP^22^, 1000 genomes project^23^, NHLBI GO Exome Sequencing Project^24^ and 69 Complete Genomics panel (http://www.completegenomics.com/public-data/69-Genomes/). Technology-specific sequencing artefacts were filtered out by using panels of BAM files of (unmatched) normal tissues containing more than 250 normal whole genomes and 500 normal whole exomes. Any somatic mutation present in at least two well-mapping reads in at least two normal BAM files was discarded. The remaining somatic mutations constituted the mutational catalogue for every matched-normal pair. The immediate 5′ and 3′ sequence context for each somatic mutation was extracted using the ENSEMBL Core APIs for human genome build GRCh37. Curated somatic mutations that originally mapped to an older version of the human genome were re-mapped using UCSC's freely available lift genome annotations tool (https://genome.htseq.org/~plantregulome/cgi-bin/hgLiftOver)^25^. Any somatic mutations with ambiguous or missing mappings were discarded from further analysis. The prevalence of somatic mutations in each sample was estimated based on a haploid human genome after all filtering was performed as previously done in ref. 12.

### Estimating the contributions of mutational signatures in each sample

The mutational catalogues of all 10,250 samples were examined in two independent and distinct steps. Initially, *de novo* extraction based on somatic substitutions and their immediate sequence context was performed to derive the set of novel consensus mutational signatures. Briefly, mutational signatures were deciphered independently for each of the 36 cancer types using our previously developed computational MATLAB framework^15^. The computational framework for deciphering mutational signatures is freely available (Supplementary Software 1) and it can be also downloaded from: http://www.mathworks.com/matlabcentral/fileexchange/38724. The algorithm deciphers the minimal set of mutational signatures that optimally explains the proportion of each mutation type found in each catalogue and then estimates the contribution of each signature to each mutational catalogue. Mutational signatures were also extracted separately for genomes and exomes. Mutational signatures extracted from exomes were normalized from the observed trinucleotide frequency in the human exome to the trinucleotide frequency of the human genome. All mutational signatures were clustered using unsupervised agglomerative hierarchical clustering and a threshold was selected to identify the set of consensus mutational signatures. Misclustering of signatures was avoided as previously described in ref. 12. A curated list of cancer census mutational signatures and their presence in human cancer can be found at our website: http://cancer.sanger.ac.uk/cosmic/signatures.

The *de novo* extraction was used to identify the complete set of consensus mutational signatures across the examined 10,250 samples. The next step of the analysis focused on accurately estimating the numbers of somatic mutations associated with each mutational signature in each sample. We usually refer to this number of somatic mutations as either the ‘contribution' of a mutational signature or the ‘exposure' to a mutational signature. Calculating the contributions of all mutational signatures was performed by estimating the number of mutations associated with the consensus patterns of the signatures of all operative mutational processes in each cancer sample. This approach allows direct comparison between cancer types, because identical signatures were used to estimate the contributions in each cancer type. More specifically, all consensus mutational signatures were examined as a set *P* containing 33 vectors 
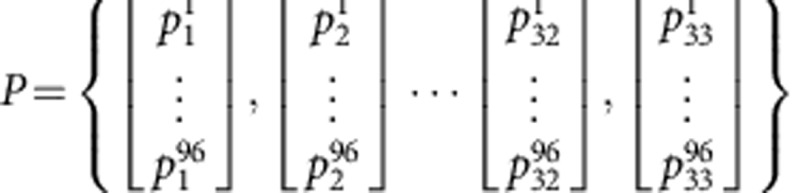
 where each of the vectors is a discrete probability density function reflecting a consensus mutational signature. The 96 non-negative components of each vector correspond to the number of mutation types (that is, somatic substitutions and their immediate sequencing context) of the consensus mutational signatures. The contributions of the mutational signatures were estimated independently for each of the 10,250 samples with a subset of consensus mutational signatures. For each sample, the estimation algorithm consists of finding the minimum of the Frobenius norm of a constrained linear function (see below for constraints) for a set of vectors *S*_1..*q*_, *q*≤33, belonging to the subset *Q*, where *Q*⊆*P* (*P* is the hitherto mentioned set encompassing all extracted consensus mutational signatures):


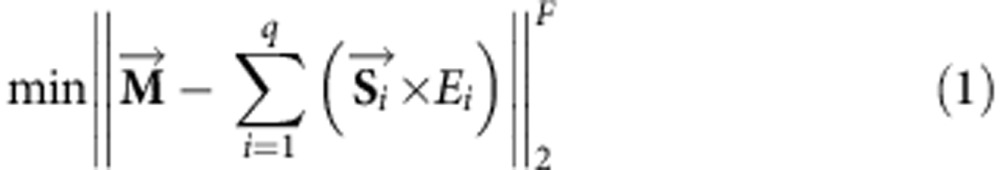


The subset *Q* is determined based on the known operative mutational processes in the cancer type of the examined sample from the mutational signature extraction process described above. For example, for any neuroblastoma sample, *Q* will contain signatures 1, 5 and 18, as these are the only known signatures of mutational processes operative in neuroblastoma^12^. In equation (1), 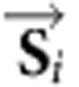 and 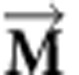 represent vectors with 96 non-negative components (corresponding to the six somatic substitutions and their immediate sequencing context) reflecting, respectively, a consensus mutational signature and the mutational catalogue of the examined sample. Hence, 
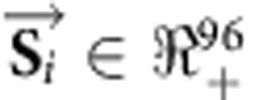
 while 
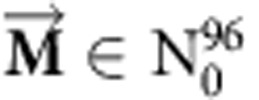
. Further, both vectors have known numerical values either from the *de novo* extraction (that is, 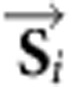) or from generating the original mutational catalogue of the sample (that is, 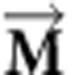). In contrast, *E*_*i*_ corresponds to an unknown scalar reflecting the number of mutations contributed by signature 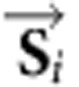 in the mutational catalogue 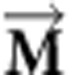.

Minimization of equation (1) is performed under several biologically meaningful linear constraints. The set of vectors in the examined set *Q* is constrained based on previously identified biological features of the consensus mutational signatures. For example, consensus signature 6 causes high levels of small insertions and/or deletions (indels) at mono/polynucleotide repeats^12^. Thus, this mutational signature will be excluded from the set *Q* when the mutational catalogue of an examined sample has only a few such indels. Similarly, there are signatures associated with other types of indels, transcriptional strand bias, dinucleotide mutations, hypermutator phenotypes and so on, and these signatures are included in the set *Q* only when the sample in question exhibits one or more of these features. Lists of features associated with different mutational signatures can be found in ref. 12 as well as at our website: http://cancer.sanger.ac.uk/cosmic/signatures. In addition to sample-specific constraints to the set *Q*, equation (1) was universally constrained in regards to the parameter *E*_*i*_. More specifically, the number of somatic mutations contributed by a mutational signature in a sample must be non-negative and it must not exceed the total number of somatic mutations in that sample. Furthermore, the mutations contributed by all signatures in a sample must equal the total number of somatic mutations of that sample. These constraints can be mathematically expressed as 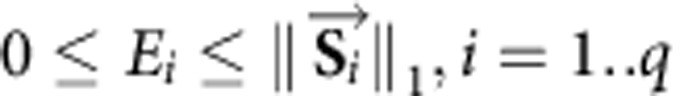, and 
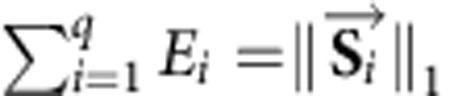
. The results for the contributions of mutational signature 3 in all 10,250 samples from the hitherto described approach are provided in Supplementary Data 1.

### Factors that influence extraction of mutational signatures

We have previously used results from simulated data to describe a plethora of factors that influence the accuracy of the extraction of mutational signatures^15^. Such factors include the number of available samples, the number of somatic mutations in a sample, the number of mutations contributed by different mutational signatures, the similarity between the patterns of the signatures of mutational processes operative in cancer samples, as well as the computational limitations of our framework. Nevertheless, in the past 3 years, our framework has proven robust and has described multiple similar and validated signatures across the spectrum of human cancer^8^,^12^,^14^,^26^,^27^,^28^,^29^,^30^,^31^.

## Additional information

**How to cite this article:** Alexandrov, L. B. *et al.* A mutational signature in gastric cancer suggests therapeutic strategies. *Nat. Commun.* 6:8683 doi: 10.1038/ncomms9683 (2015).

## Supplementary Material

Supplementary Data 1 List of examined cancer samples with their respective cancer types, sequencing types, accession numbers, source from which the data were taken, and mutations attributed to signature 3.

Supplementary Data 2 Mutational signatures in all examined gastric samples.

Supplementary Data 3 Indels and structural variants in whole-genome sequenced gastric cancers.

Supplementary Data 4 Indels in whole-exome sequenced gastric cancers.

Supplementary Data 5 Examined gastric cancer cell lines.

Supplementary Software 1 MATLAB based computational framework for deciphering signatures of mutational processes.

## Figures and Tables

**Table 1:** Presence of signature 3 and gastric cancer histology.

	**Total samples**	**Presence of signature 3**		**Fisher's exact test's two-tailed** ***P*** **value**
		**Present (%)**	**Absent (%)**	
**Whole-genome sequencing cohort**	100	12 (12.0)	88 (88.0)	
Compact discohesive growth pattern				0.0003
Present	11	6 (54.5)	5 (45.5)	
Absent	89	6 (6.7)	83 (93.3)	
				
**Whole-genome sequencing and TCGA cohorts combined**	409	37 (9.0)	372 (91.0)	

Laurens' tumour type*				
Intestinal	271	32 (11.8)	239 (88.2)	0.0058†
Diffuse	105	2 (1.9)	103 (98.1)	0.0015‡
Mixed	33	3 (9.1)	30 (90.9)	

TCGA, The Cancer Genome Atlas.

^*^Cases in the TCGA cohort without information on Laurens' tumour type are excluded for analysis.

^†^Intestinal type versus diffuse type and mixed type.

^‡^Diffuse type versus intestinal type and mixed type.
