# Supplementary material for: A mutational signature in gastric cancer suggests therapeutic strategies
Source: Nat Commun. 2015 Oct 29;6:8683. doi: 10.1038/ncomms9683 (PMC4918743; doi:10.1038/ncomms9683)
Supplement: Supplementary Software 1 — MATLAB based computational framework for deciphering signatures of mutational processes. [file ncomms9683-s6.zip › Supplementary Software 1/readme.pdf]

FRAMEWORK FOR DECIPHERING SIGNATURES OF MUTATIONAL PROCESSES FROM A SET OF MUTATIONAL CATALOGUES OF CANCER GENOMES

July 19, 2013

INTRODUCTION

The purpose of this document is to provide brief but essential guide for using the Wellcome Trust Sanger Institute (WTSI)'s framework for deciphering signatures of mutational processes from catalogues of cancer genomes. Detailed explanation of the theoretical model and the framework is available in our manuscript entitled "Deciphering signatures of mutational processes operative in human cancer" by Alexandrov *et al.*, Cell Reports, Volume 3, Issue 1, 246-259:

http://www.cell.com/cell-reports/fulltext/S2211-1247(12)00433-0

In addition to the framework's source code, four examples and two input files are provided to better illustrate how the framework could be applied to mutational catalogues of cancer genomes.

PREREQUISITES

The framework is written in MATLAB and requires the following packages with the specified (or newer) versions:

```
MATLAB                          Version 7.7   (R2008b)
Parallel Computing Toolbox      Version 4.0   (R2008b)
Bioinformatics Toolbox          Version 3.2   (R2008b)
Optimization Toolbox            Version 4.1   (R2008b)
Statistics Toolbox              Version 7.0   (R2008b)
```

Please note that MATLAB and the parallel toolbox are essential for running the framework's core functionality. The other three toolboxes are desirable but the code for deciphering mutational signatures could be executed without them as other freely available packages have been leveraged in places.

Accurately deciphering signatures of mutational processes is computationally intensive and the framework is usually run on a cluster (or a farm) with at least 100 nodes. Further, the four provided examples make the assumption that the default matlabpool has already been preconfigured (please refer to MATLAB's documentation for configuring matlabpool) and they will use all available labs for the default matlabpool configuration.

By default, the framework uses the nonnegative matrix factorization (NMF) solver from (Brunet *et al.*, PNAS, 2004, 12, 4164-4169), which is based on the multiplicative update algorithm (Lee and Seung, 1999, Nature 401, 788-791). However, if the Statistics Toolbox is available, the provided NMF solver (*i.e.*, ***nnmf***) could be used instead and it generally produces faster results. Additional freely available NMF solvers based on multiplicative update and other algorithms are provided. However, in general all solvers (with the appropriate options) converge to almost identical solutions and the main difference between them is CPU execution time and the required memory for execution.

FOLDER STRUCTURE

The framework contains six folders, four example files, and this readme file. The source folder contains all code related to deciphering signatures of mutational processes including several nonnegative matrix factorization solvers. The plotting folder contains all source code related to plotting mutational signatures (with and without strand bias) as well as a plot that could be used for identifying the number of operative mutational signatures. The input folder contains MATLAB (*i.e.*, *.mat) files for the given examples each containing a set of mutational catalogues of cancer genomes (see below). The output folder contains MATLAB (*i.e.*, *.mat) files each with the results of executing the framework. The framework uses the temp folder for temporary storage during execution. The data folder contains additional mutational catalogs that could be analyzed using this framework. The four provided examples are discussed in further details in next sections.

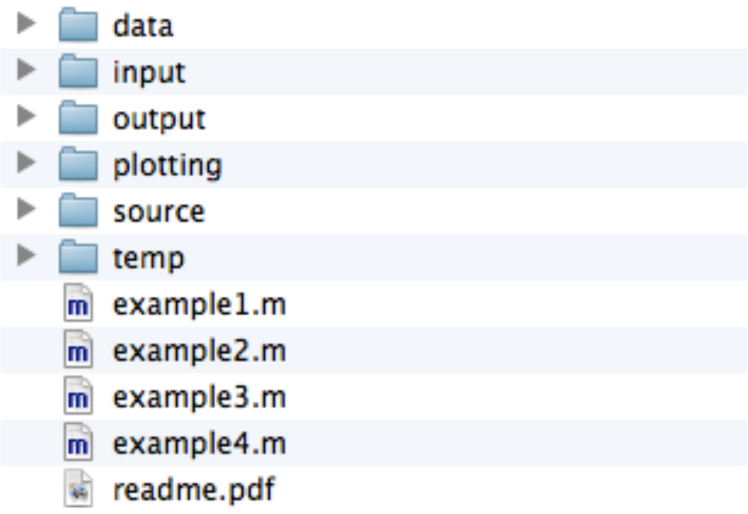

INPUT FILE FORMAT

An input file is a MATLAB (*i.e.*, *.mat) file that contains a set of mutational catalogues and metadata information about the cancer type, and the mutational types, subtypes, *etc.* for which these mutational catalogues have been defined. For example, the provided **21_WTSI_BRCA_whole_genome_substitutions.mat** is shown in the Figure of this section. The file contains the following fields:

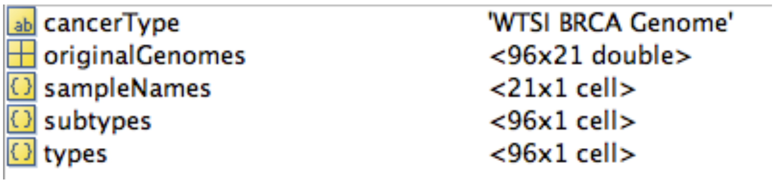

- **cancerType –** string describing the type of samples in the file.

- **sampleNames –** a list of strings in which each element corresponds to the name of the analyzed sample.
- **types –** a list of strings in which each element corresponds to the name of the mutational types for which the catalogues have been defined.
- **subtypes –** a list of strings in which each element corresponds to the name of the mutational subtype for which the catalogues have been defined. Note that additional fields could be added if more classes of mutational types are being examined (*e.g.,* strand bias).
- **originalGenomes** – an array containing mutational catalogues of cancer genomes with size <samples> by <mutational types> in which each element corresponds to the number of mutations per sample per mutational type (and its subtype, *etc.*).

Please note that an input file could contain more fields but the fields above are required for the framework to process the provided mutational catalogues.

DESCRIPTION OF PROVIDED EXAMPLES
The provided examples perform 10 iterations per available core. Please note that there is an expectation of at least 1,000 iterations (*i.e.,* 100 available cores) and this number should be adjusted accordingly to the available nodes, otherwise it is possible that the identified mutational signatures are not completely accurate. Further, each of the examples plots the result

example1.m: This example illustrates deciphering mutational signatures from a set of mutational catalogues derived from 21 breast cancer genomes, when the number of signatures is known.

example2.m: This example illustrates identifying the number of mutational processes operative in a set of mutational catalogues derived from 21 breast cancer genomes.

example3.m: This example illustrates reading a previously saved output file and plotting the data. The example relies on the output of example1.m.

example4.m: This example illustrated deciphering mutational signatures with a third mutational subtype (*i.e.,* strand bias) from a set of mutational catalogues derived from 100 breast cancer exomes.

ADDITIONAL DATA
Recently, we performed a large-scale mutational signature analysis of the mutational catalogues of 7,042 primary cancers from 30 different classes. These and other data have been included (as ready to use MATLAB input files) in the *data* folder to allow results reproducibility as well as new global scale analysis of mutational signatures. The data folder will be periodically updated to include additional mutational catalogs from newly sequenced cancer samples. Currently, the data folder is organized by publications in which the framework has been leveraged.

COPYRIGHT

This software and its documentation are copyright 2013 by the Wellcome Trust Sanger Institute/Genome Research Limited. All rights are reserved. This software is supplied without any warranty or guaranteed support whatsoever. Neither the Wellcome Trust Sanger Institute nor Genome Research Limited is responsible for its use, misuse, or functionality.

CONTACT INFORMATION

Please address any queries or bug reports to Ludmil B. Alexandrov at la2@sanger.ac.uk.
